# The role of calcium-calpain pathway in hyperthermia

**DOI:** 10.3389/fmmed.2022.1005258

**Published:** 2022-10-10

**Authors:** Atsushi Enomoto, Takemichi Fukasawa

**Affiliations:** ^1^ Laboratory of Molecular Radiology, Center for Disease Biology and Integrative Medicine, Graduate School of Medicine, The University of Tokyo, Tokyo, Japan; ^2^ Department of Dermatology, Graduate School of Medicine, The University of Tokyo, Tokyo, Japan

**Keywords:** calcium, calpain, hyperthermia, autophagy, cell death

## Abstract

Hyperthermia is a promising anticancer treatment modality. Heat stress stimulates proteolytic machineries to regulate cellular homeostasis. Calpain, an intracellular calcium (Ca^2+^)-dependent cysteine protease, is a modulator that governs various cellular functions. Hyperthermia induces an increase in cytosolic Ca^2+^ levels and triggers calpain activation. Contrastingly, pre-exposure of cells to mild hyperthermia induces thermotolerance due to the presence of cellular homeostatic processes such as heat shock response and autophagy. Recent studies suggest that calpain is a potential key molecule that links autophagy and apoptosis. In this review, we briefly introduce the regulation of intracellular Ca^2+^ homeostasis, basic features of calpains with their implications in cancer, immune responses, and the roles and cross-talk of calpains in cellular protection and cell death in hyperthermia.

## Introduction

Hyperthermia is a well-known cancer treatment method which affects tissues and cells in various ways. Hyperthermia increases vascular permeability, elevation of blood flow, and thus oxygenation in the tumor, which sensitizes tumor cells to chemotherapy and radiotherapy (Wust et al., 2002; Issels 2008). Hyperthermia also affects multiple cellular processes, like e.g., cell survival, immune responses, and cell death (Zhang et al., 2008; Kassis et al., 2021). It increases cell temperature and induces many biochemical changes, such as generation of reactive oxygen species, increase in intracellular calcium ion concentration, and protein degradation (Roti Roti, 2008; Hou et al., 2014). Hyperthermia-induced protein denaturation, aggregation, or degradation is a key event in the disruption of cellular homeostasis (Luo et al., 2000; Ahmed et al., 2020). Intracellular protein degradation is regulated by multiple proteolytic pathways, including lysosome-, calcium-, and proteasome-dependent mechanisms (Goll et al., 2003; Ciechanover, 2005). However, the molecular mechanisms underlying thermal protein degradation, their roles, and their cross-talk in thermal killing are largely unknown. In this mini review, we focused on the involvement and contribution of calpain in hyperthermia-induced cell death.

## Cellular calcium signaling

The human body maintains Ca^2+^ concentration in plasma at approximately 2 mM regardless of the site. On the contrary, the intracellular Ca^2+^ concentration is maintained at 100–200 nM, and the difference between intracellular and extracellular Ca^2+^ concentrations is more than 10,000-fold. Several mechanisms are involved in Ca^2+^ influx from outside the cell, including voltage-gated channels in the plasma membrane, ligand-operated channels such as those found in NMDA receptors, and Ca^2+^/Na^+^ exchange transporters that utilize the concentration gradient between the intracellular and extracellular environment (Brini et al., 2014; Woll and Van Petegem, 2022). However, elevated intracellular Ca^2+^ concentrations are also cytotoxic and are therefore immediately effluxed; Ca^2+^ influx is through a channel, whereas efflux is *via* a pump. The Ca^2+^-ATPase present in the plasma membrane and endoplasmic reticulum (ER) membrane acts as a pump translocating Ca^2+^ from the cytosol. The ER stores Ca^2+^ taken up by this Ca^2+^ pump (sarco (endo)plasmic reticulum Ca^2+^/Mg^2+^-ATPase) (Primeau et al., 2018). Contrastingly, the efflux system from the ER to the cytoplasm contains inositol 1, 4, 5-triphosphate (IP_3_)-induced Ca^2+^ release and Ca^2+^-induced Ca^2+^ release. IP_3_-induced Ca^2+^ release liberates Ca^2+^ from the IP_3_ receptor (IP_3_R) of the ER upon IP_3_ stimulation, while Ca^2+^-induced Ca^2+^ release leads to Ca^2+^ release into the cytosol when Ca^2+^ concentration in the surrounding ER increases (Fabiato, 1983; Foskett et al., 2007). The golgi imports Ca^2+^ using a secretory pathway Ca^2+^ ATPase pump and releases it through a channel sensitive to IP_3_Rs. Thermal treatment of cells induces an increase in intracellular Ca^2+^ (Kameda et al., 2001; Enomoto et al., 2022). Mild thermotolerance protects cells against heat-induced Ca^2+^ release (Bettaieb and Averill-Bates., 2015). Heat-induced Ca^2+^ burst is brought about by the activity of Ca^2+^ uptake into the ER by sarco (endo) plasmic reticulum Ca^2+^ ATPase and changes in the channel opening probability of IP_3_R (Itoh et al., 2014). Additionally, hyperthermia increases phosphorylation of IP_3_R (Li et al., 2014).

Moreover, close contact between the ER and mitochondria has recently been shown to play an important role in the control of Ca^2+^ homeostasis (Lim et al., 2021). At the mitochondria-ER contact sites, Ca^2+^ is transferred from the ER directly to the mitochondria through a protein complex including IP_3_R, voltage-gated anion channel 1, and mitochondria calcium uniporter.

In the mitochondria, Ca^2+^ influx is mediated by the mitochondria calcium uniporter. Mitochondria calcium uniporter does not uptake Ca^2+^ when the Ca^2+^ concentration outside the mitochondria is low, but uptake Ca^2+^ when the extracellular concentration outside the mitochondria is high (Finkel et al., 2015). Excess Ca^2+^ uptake into the mitochondria *via* calcium uniporters induces an increase in the permeability of the mitochondrial inner membrane (permeability transition), which triggers the release of cytochrome *c*, leading to caspase activation and apoptosis (Li et al., 2014). Ca^2+^ is released from the mitochondria through Na^+^/Ca^2+^/Li^+^ exchangers and mitochondrial permeability transition pore.

## Structure of calpain

Calpains are calcium-activated neutral proteases that catalyze the cleavage of various proteins, including enzymes, transcription factors, and cytoskeletal proteins, in many mammalian tissues (Goll et al., 2003). There are currently 15 known human calpain isoform genes. They can be classified according to their localization (ubiquitous or tissue-specific). The most intensely studied members of the calpain family are calpain-1 and -2, which are heterodimers that consist of a large catalytic subunit and a small regulatory subunit (Campbell and Davies, 2012). The catalytic subunit can be divided into four functional domains (DI-DIV), while the small subunit has two domains (DV-DVI). DI is autolyzed during activation. The protease domain (DII) contains the active site of catalytic triad residues. DIII has a β-sandwich structure that is similar to the C2 domain. This C2-like domain binds Ca^2+^ and may have a role in the activation of calpain. DIV and DVI contain five EF-hand motifs that are involved in the dimerization of the catalytic and regulatory subunits (Blanchard et al., 1997). DV is the amino-terminal end of the regulatory subunit and contains clusters of glycine residues. This domain is thought to have an important role in membrane anchoring.

## Activation mechanism of calpain

Calpain exists as an inactive proenzyme in the cytosol. Increase in cellular Ca^2+^ level causes translocation of calpain to the membrane, where it becomes active. During activation, the autolysis of the DI domain and dissociation of 30 K from 80 K takes place. A proposed mechanism for activation comprises two steps (Khorchid and Ikura, 2002; Suzuki et al., 2004). The first step is the release of constrains imposed by domain interactions. Binding of Ca^2+^ to domains III, IV, and VI, leads to the weakening of the interaction between DIII and DII and abrogation of the interaction between the N-terminal α-helix of DI and the second EF-hand motif of DVI, releasing DI from DVI. The second step of activation involves realignment of the catalytic core into its active state. Binding of Ca^2+^ to the protease domain brings IIa and IIb close together to form a catalytic site. Hyperthermia has been shown to stimulate calpain activity (Bettaieb and Averill-Bates, 2015). Correspondingly, calpain was cleaved to generate its active form and expression of calpain-1 and/or calpain-2 increased in heat-treated cells (Somwaru et al., 2004; Hou et al., 2014).

Calpastatin (CAST), a ubiquitously expressed endogenous calpain inhibitor, suppresses calpain-1 and -2 (Hanna et al., 2008). Initial studies established that this inhibitor is a heat-stable protein, and it has subsequently been shown that it is resistant to numerous denaturing agents such as urea, sodium dodecyl sulfate, and trichloroacetic acid (Geesink et al., 1998). CAST binds the penta EF-hand domains in the catalytic large subunits and regulatory small subunits of calpain across the active site to sterically hinder substrate access. CAST is regulated by phosphorylation modifications. Phosphorylation of CAST by Protein kinase A can modify its subcellular localization and repress its activity (Averna et al., 2001). It is cleaved by caspases during apoptosis (Kato et al., 2000). Ectopic expression of testis-specific CAST in pachytene spermatocytes suppressed heat-induced apoptosis of germ cells (Somwaru et al., 2004).

## Calpain substrates associated with cell survival and death

Various calpain substrates are associated with cell death and survival (Shapovalov et al., 2022). Several studies have demonstrated that calpains interact with the caspase family of cysteine proteases to initiate apoptosis. Proteolytic cleavage by calpains directly activates caspase-7, -10, and -12 (Nakagawa and Yuan, 2000; Gafni et al., 2009). Calpain can facilitate apoptosis through the cleavage of the proapoptotic BCL-2 family members. For example, calpain-1 and -2 mediated cleavage of Bcl-2-associated X protein promotes cytochrome *c* release (Gao and Dou, 2021).

Calpain has been implicated in pro-survival activities. Cleavage of the tumor suppressor p53 by calpain attenuates apoptosis (Gonen et al., 1997). Calpain can promote cell survival by activation of NF-κB by proteolysis of its inhibitor IκBα (Han et al., 1999). Moreover, calpain promotes cell survival by AKT-FOXO signaling (Ho et al., 2012).

## Implications of calpain in cancer

Many research groups have reported that aberrant expressions of calpains are associated with cancer progression (Shapovalov et al., 2022). For example, transcriptional level of *CAPN1* is associated with higher regional metastasis in renal cell carcinoma (Braun et al., 1999). High expression of calpain-2 is associated with resistance to platinum-based therapies (Storr et al., 2012a). However, there are several conflicting evidences suggesting the anti-tumorigenic roles of calpains. Tumors of the bile ducts and ampulla with low calpain-1 and -2 are more aggressive in pancreatic carcinoma (Storr et al., 2012b). It has been observed that expression levels of calpain mRNA and protein do not necessarily correlate. For example, expression level of *CAPN1* mRNA is significantly higher in basal cell skin carcinoma than in normal tissue, while the protein level of calpain-1 is reduced, probably due to higher proteolytic and autolytic activity (Reichrath et al., 2003). Calpain activity is also regulated through phosphorylation by kinases (Shiraha et al., 2002; Glading et al., 2004). Thus, in addition to the expression levels of calpains, their activities and the kinetics of their substrates should be investigated for further understanding of its roles in cancer.

## Calpain and immune response

Calpains are involved in activities of immune cells. Expression of calpain is particularly low in resting lymphocytes, but synthesis and secretion of calpain occurs in active lymphocytes (Deshpande et al., 1995). Inhibition of calpain reduces proliferative efficiency of T cells (Smith et al., 2011). Calpain-mediated degradation of IκB, an inhibitor of NF-κB, is important in T-cell activation (Schaecher et al., 2004). Calpain regulates secretion of cytokines. Inhibition of calpain could attenuate the secretion of multiple cytokines, including IL-6, IL-12, IL-17, IFN-γ and TNF-α (Smith et al., 2011). Calpain also participates in migration and adhesion of inflammatory cells by modulating integrin-mediated functions through cleavage of integrin-binding partners or by altering the levels of adhesion molecules such as intracellular cell adhesion molecule-1 (ICAM-1) (Cuzzocrea et al., 2001; Stalker et al., 2005; Zhao et al., 2012).

Hyperthermia with 39°C–42°C has been shown to stimulate and amplify a broad array of immune responses. Hyperthermia induces the release of a number of cytokines such as IL-2, IL-6, IL-8, IFN-γ and TNF-α (Takahashi et al., 2012). These cytokines stimulate differentiation of T lymphocytes and B-cells, maturation of DC cells, activation of T cells or macrophages, and amply other immune responses (Dinarello 2007). Heat upregulates the expression of L-selectin, ICAM-1 on high endothelial venules (Wang et al., 1998; Chen et al., 2009), and increases the integrin- and selectin-mediated adhesion of lymphocytes to the endothelium, which enhances immune cell recruitment and then infiltration to the tumor microenvironment (Evans et al., 2000; Shah et al., 2002).

Together, it is suggested that calpain and hyperthermia have a common point of action in the immune responses. Calpain might participate and act in multiple discrete immune response steps that is activated by hyperthermia. More research is needed to elucidate whether and how calpain is involved in hyperthermia-stimulated immunity.

## Calpain and hyperthermia

**FIGURE 1:**
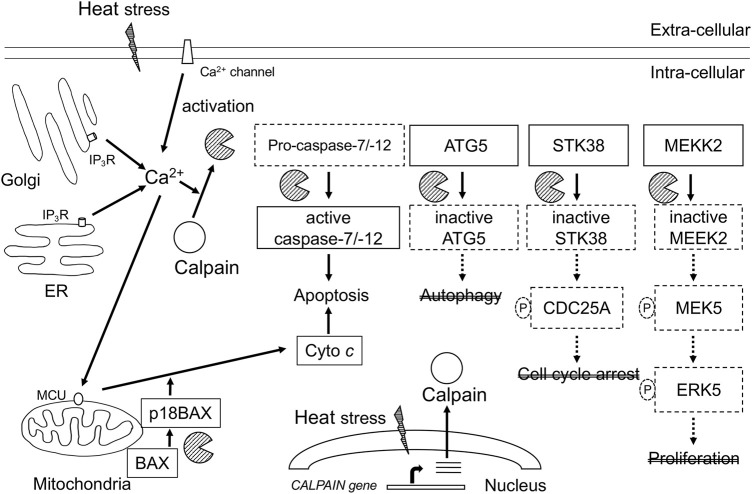
Schematic representation of calcium-calpain dependent death signaling in hyperthermia. Calpains are activated by increase in Ca^2+^ intracellular concentration through Ca^2+^ influx from intracellular stores and Ca^2+^ entry from the extracellular fluid. Calpains are implicated in multiple key signaling pathways associated with stress response, cell death, and cell survival. ER, endoplasmic reticulum; IP_3_R, 1, 4, 5-triphosphate (IP_3_)-receptor; MCU, mitochondrial calcium uniporter; Cyto *c*, cytochrome *c*; ATG5, autophagy related gene 5; STK38, serine-threonine kinase38; MEKK2, MEK/ERK kinase 2.

Hyperthermia induces the activation of calpains and cleavage CAST at 42°C–43°C (Bettaieb and Averill-Bates, 2015). Calpain-mediated proteolysis of cell signaling regulators has been reported in response to hyperthermia. Diverse studies have shown that the impact of calpains in hyperthermia occurs at multiple levels, as summarized in Figure 1. We demonstrated that hyperthermia decreases the expression and kinase activity of several MAP3K members (such as TAK1 and MEKK2) by calpain-mediated degradation (Enomoto et al., 2022). TAK1 and MEKK2 are pivotal activators of MAPK signaling and control cell viability and inflammation by activating downstream effectors such as NF-κB, JNK, and ERK5, suggesting that they promote cell survival by regulating apoptosis (Takaesu et al., 2003; Kesavan et al., 2004). Therefore, hyperthermia can attenuate NF-κB activity by decreasing upstream kinases TAK1 and MEKK2 despite cleavage of its inhibitor IκBα by calpain. Calpain-mediated cleavage of STK38 was also observed in heat-treated HeLa cells (Enomoto et al., 2019). STK38, a member of the AGC kinase family, is involved in the regulation of centrosome duplication and DNA damage-induced G2/M checkpoint (Hergovich et al., 2007; Fukasawa et al., 2015). Together, hyperthermia-induced degradation of cell signaling regulators may be incapable of transducing signals and/or activating each downstream target by phosphorylation, leading to the inhibition of cell survival and anti-apoptotic or DNA-damage responses, thereby contributing to thermal killing. Thus, hyperthermia may be useful in combination with radiation therapy.

Calpain can cleave target proteins, leading to cell rupture. A curious role of calpain in apoptosis is the caspase/calpain cascade cross-talk. Calpain is an activator of caspase-7 and -12 (Nakagawa and Yuan, 2000; Gafni et al., 2009). In addition, caspase-3 cleaves calpastatin, resulting in the activation of calpain (Pörn-Ares et al., 1998). Thus, calpain plays an active role in the activation of caspases and apoptosis. Incidentally, several synthetic calpain inhibitors prevent heat-induced apoptosis (Lizama et al., 2009). Knock down of calpain-1 or -2 inhibited heat-induced apoptosis of U2OS cells (Hou et al., 2014). Interestingly, thermal tolerance due to mild hyperthermia protected against heat-induced activation of calpain and apoptosis (Bettaieb and Averill-Bates., 2015). These reports suggest that activated calpain is a potential indicator of cellular heat sensitivity.

Autophagy is a conserved and highly regulated process of the lysosomal pathway that cleanses cells by recycling damaged proteins, macromolecules, and organelles (Dikic and Elazar, 2018). Autophagy acts as a pro-survival or pro-death mechanism, depending on cell types, context, and kind of stimulus. Hyperthermia at 40°C–43°C induces autophagy; in most cases, triggering of this pathway was associated with the promotion of cell survival (Zhao et al., 2009; Liu et al., 2010). Exposure of cells to mild heat (42°C for ≤1 h) increased levels of autophagy proteins such as ATG7 (autophagy-related gene 7) and ATG12/ATG5 conjugate, and thermotolerant cells showing higher expression levels of these autophagy proteins were resistant to long-term heat stress-induced (42°C for 2–3 h) apoptosis (Kassis et al., 2021). Inhibition of autophagy with bafilomycin may increase cell death by hyperthermia (Komata et al., 2004). Several evidences indicate that calpain is involved in autophagy. Calpain cleaves the α-subunit of heterotrimeric G proteins (G_Sα_), leading to a reduction of autophagy initiation (Rivero-Ríos et al., 2016). ATG5, which is required for the formation of autophagosomes, is cleaved and inactivated by calpains. Interestingly, calpain-cleaved ATG5 was shown to translocate to the mitochondria and induce apoptosis by blocking the antiapoptotic function of Bcl-xL (Yousefi et al., 2006). Additionally, knockdown of ATG5 enhanced heat-induced apoptosis (Jiang et al., 2019). Once calpain is activated, it may switch autophagy to apoptosis (Nakagawa and Yuan, 2000; Yousefi et al., 2006). Thus, calpain is a potential key molecule that links autophagy and apoptosis in hyperthermia.

## Conclusion

Calpains participate in a variety of physiological processes, including cell proliferation and death, immune responses, and are involved in tumorigenesis (Goll et al., 2003; Shapovalov et al., 2022). However, their regulatory mechanism in pro-survival and cell death signaling pathways are largely unknown. Contrastingly, hyperthermia induces cell death directly or indirectly through activation of immune sytems, but under certain conditions, causes transient heat resistance (called thermotolerance) through cellular homeostatic processes such as heat shock responses and autophagy (Ahmed et al., 2020; McCormick et al., 2021). The efficiency of hyperthermia depends on the temperature and duration of heat treatment. Inhibition of autophagy sensitizes cells to heat (Komata et al., 2004; Kassis et al., 2021). Thus, not only is activation of the Ca^2+^-calpain signaling pathway required, but also appropriate conditions are needed for calpain to act as a switch between protective autophagy and apoptosis in hyperthermia. Further studies should be conducted to explore the regulatory mechanism of calpain in hyperthermia.
